# Integrated portable ECG monitoring system with CNN classification for early arrhythmia detection

**DOI:** 10.3389/fdgth.2025.1535335

**Published:** 2025-03-18

**Authors:** Aayush Panwar, Modigari Narendra, Arnav Arya, Rohan Raj, Arnab Kumar

**Affiliations:** ^1^ School of Electronics Engineering, Vellore Institute of Technology, Chennai, India; ^2^ School of Computer Science and Engineering, Vellore Institute of Technology, Chennai, India

**Keywords:** ECG, portable monitoring, convolutional neural networks, arrhythmia detection, Arduino Nano, AD8232 ECG sensor

## Abstract

**Introduction:**

Electrocardiograms (ECGs) play a crucial role in diagnosing heart diseases by capturing the electrical activity of the heart. With the rising need for real-time cardiac monitoring, portable solutions have gained significance for timely detection and intervention. This study presents a portable ECG monitoring system incorporating Convolutional Neural Networks (CNNs) for accurate classification of cardiac abnormalities, including arrhythmias.

**Methods:**

The proposed system consists of an Arduino Nano microcontroller interfaced with an AD8232 ECG sensor for real-time ECG signal acquisition. The collected ECG data undergoes preprocessing before being fed into CNN models trained on the MIT-BIH Arrhythmia dataset. The model is designed for both binary and multi-class classification, distinguishing normal and abnormal heart rhythms. Performance metrics, including accuracy, were evaluated against state-of-the-art approaches to assess classification effectiveness.

**Results:**

Experimental evaluations demonstrate the CNN model’s high classification accuracy, achieving 98.35% in binary classification and 99.3% in multi-class classification. These results surpass existing benchmarks, highlighting the efficiency of the proposed system. The system's low-cost hardware and real-time classification capabilities enhance its suitability for continuous cardiac monitoring.

**Discussion:**

The proposed ECG monitoring system presents a reliable and cost-effective solution for early arrhythmia detection. By leveraging CNNs, it ensures accurate classification of cardiac abnormalities, making it a promising tool for both clinical and remote healthcare settings. Its potential impact extends to real-time monitoring, early diagnosis, and personalized healthcare, contributing to improved cardiovascular health management.

## 1 Introduction

The ECG is an important tool for identifying heart diseases as it offers information about the heart's electrical activity. Patterns in ECG signals helps in the understanding and diagnosis of various cardiac diseases. Accurate and prompt interpretation of these signals is critical for early detection and treatment of heart disorders, highlighting the value of ECG monitoring in clinical practice. Changes in medical care, such as prevention and personalized medicine, underline the need of portable ECG monitoring. The traditional pattern of hospital visits for ECG tests might be too expensive, particularly for those with chronic diseases or who require ongoing treatment. Recent technological advances have resulted in more efficient ECG monitoring solutions.

**Figure 1:**
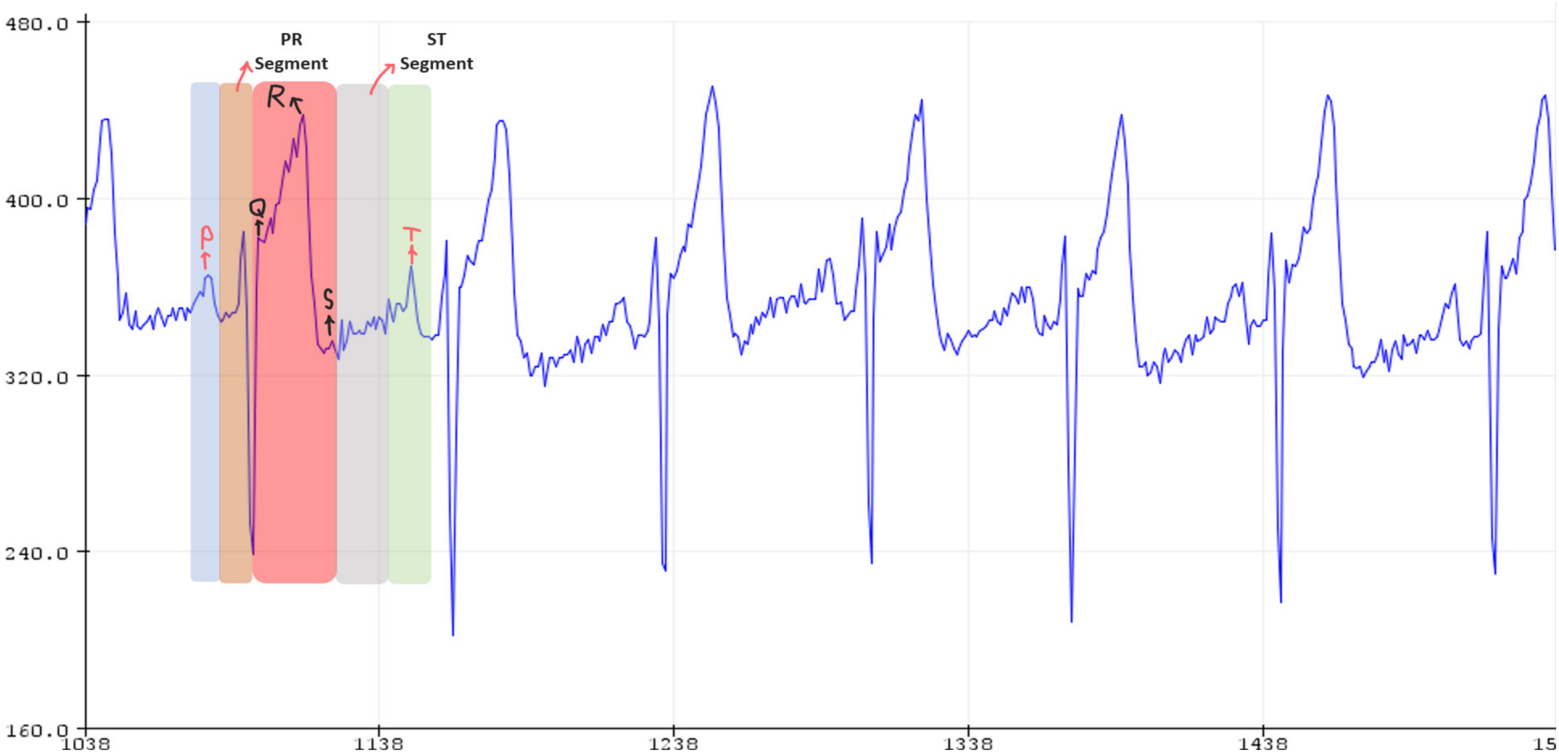
Received signal from ECG device, image obtained from cited Database.

The fundamental elements of ECG patterns are known as P, QRS and T waves, each displaying either positive or negative voltage (1). These signals arise from the electrical activity associated with the contraction and relaxation phases of distinct regions within the heart muscle. A sample ECG signal signal I shown in Figure 1. Each of the waves and segments is explained in detail below:

• The P wave is typically indicating an ectopic atrial pacemaker. If the P wave is of unusually long duration, it may represent atrial enlargement. Typically, a large right atrium gives a tall, peaked P wave while a large left atrium gives a two-humped bifid P wave.
• The PR interval is measured from the beginning of the P wave to the beginning of the QRS complex. This interval reflects the time the electrical impulse takes to travel from the sinus node through the atrioventricular (AV) node. A PR interval shorter than 120 ms suggests that the electrical impulse is bypassing the AV node, as in Wolff-Parkinson-White syndrome. A PR interval consistently longer than 200 ms diagnoses first degree atrioventricular block. The PR segment (the portion of the tracing after the P wave and before the QRS complex) is typically completely flat, but may be depressed in pericarditis.
• The electrical current that contracts the right and left ventricles is called the QRS complex. If the QRS complex is wide (longer than 120 ms) it suggests disruption of the heart's conduction system, such as in Left Bundle Branch Block (LBBB), Right Bundle Branch Block (RBBB), or ventricular rhythms such as ventricular tachycardia. Metabolic issues such as severe hyperkalemia, or tricyclic antidepressant overdose can also widen the QRS complex. An unusually tall QRS complex may represent left ventricular hypertrophy while a very low-amplitude QRS complex may represent a pericardial effusion or infiltrative myocardial disease.
• ST—The ST segment connects the QRS complex and the T wave; it represents the period when the ventricles are depolarised. It is usually isoelectric, but may be depressed or elevated with myocardial infarction or ischemia. ST depression can also be caused by Left Ventricular Hypertrophy (LVH) or digoxin. ST elevation can also be caused by pericarditis, Brugada syndrome, or can be a normal variant.
• The T wave represents the repolarisation of the ventricles. Inverted T waves can be a sign of myocardial ischemia, left ventricular hypertrophy, high intracranial pressure, or metabolic abnormalities. Peaked T waves can be a sign of hyperkalemia or very early myocardial infarction.

Portable electrocardiogram systems have gained attention for their potential in enabling convenient cardiac monitoring. They help users to instantly monitor their heart status wherever they are. The existing studies have explored diverse methodologies and technologies aimed at enhancing accuracy, classification and signal transmission capabilities of these systems. Numerous studies have been conducted to study various methodologies and technologies aimed at enhancing the precision, categorization, and signal transmission capabilities of portable ECG monitoring systems. Among other things, researchers have built complete systems for monitoring ECG signals in real time via the Internet, low-cost Holter systems based on microcontrollers, and portable ECG recorders that connect to cellphones and other Bluetooth-enabled devices.

The primary motivation for our project is to solve critical difficulties in cardiovascular health, particularly the early diagnosis and management of arrhythmias. Arrhythmias are a major health concern worldwide, with potentially fatal implications if undiagnosed or mistreated. Our project aims to address this issue by creating an integrated portable ECG monitoring device supplemented with cutting-edge machine learning algorithms. By creating an integrated portable ECG monitoring system with CNN classification for early arrhythmia identification, we hope to make a real difference in the lives of patients, healthcare providers, and communities around the world.

## 2 Literature survey

Kong et al. (2) described a comprehensive method for real-time ECG signal monitoring via the Internet. The system consists of three main components: a portable Holter device with dual-channel ECG readings, an FM transmitter for live data feed, and a CompactFlash slot for storage. The Holter, which is based on an 8,051 microprocessor, can run for more than 24 h on two AA rechargeable batteries. Segura et al. (3) created a low-cost, microcontroller-based Holter system for ambulatory ECG capture, taking use of the availability of powerful but affordable computers. The methodology follows a logical sequence, beginning with ECG signal acquisition via three strategically placed electrodes and progressing through signal processing steps such as amplification, Analog to Digital Converter (ADC), and data storage.

Chiang et al. (4). enhanced ECG signal processing for heart rate regulation by integrating various techniques, including a biomedical signal amplifier/filter, protection circuit, ADC, USB flash disk and computer interface, to create a portable ECG recorder. Using the MSP430F149 as the master CPU and SL81 IHS as the interface chip, the system's performance is managed and lead II ECG signals are collected. MathWorks’ computer language is employed to design a program locating R-wave peaks. Sung-Yuan et al. (5). designed an Android smartphone and Bluetooth-enabled device (HL-MD08R-C2 module) for ECG signal acquisition, utilising a conductive belt and KY202 sensor. The captured signal is transmitted to the smartphone, displayed on-screen for real-time monitoring. Tomcat an open-source servlet is used to run Java based program for Managing the system on HTTP web server. Operating on a Hadoop distribution system.

Campillo et al. (6). used MSP430F5419A and CC2540 microcontrollers, integrating GSM/GPRS and GPS modules. The device features a TFT display showcasing critical parameters, including signal strength, battery status, time, Bluetooth connectivity and connection status. Powered by a Li-Ion battery, the portable system ensures 48 h of continuous ECG signal acquisition. Celebi et al. (7). used two independent circuits, each powered by small batteries for compactness, form the basis of a system designed to detect heart signals and beats. The compact size of the circuits allows for portability. The system operates autonomously without reliance on external platforms such as smartphones or PCs. Users can observe real-time heart rate results displayed directly on the device. The absence of mention regarding ML models suggests a non-automated approach, focusing on simplicity and direct signal display for user awareness.

Hodrob et al. (8). proposed healthcare system integrating an Android app for customers and a web application for doctors, utilising IoT technology. A Raspberry Pi, equipped with sensors from HealthyPi HAT and HealthyPi, captures signals, sends data to a cloud server and applies data mining and ML models for analysis. Users access analysed data via the mobile app, while doctors use the web portal. Achieving a 99.3% accuracy, addressing the critical challenge of ensuring a reliable real-time system for healthcare applications. Security measures are prioritised to safeguard sensitive health data. In their study, Gradl et al. (9) present an Android ECG monitoring application which utilises the Pan-Tompkins algorithm to accurately detect QRS complexes and employs additional algorithms to identify abnormal heartbeats, boasting an accuracy rate exceeding 99%. The application seamlessly interfaces with external ECG sensors through Bluetooth.

Jeon et al. (10) introduced a portable ECG device designed to facilitate the early detection of atrial fibrillation (AF) and myocardial ischemia, both critical for enhancing survival rates among cardiac patients and able to achieve sensitivity rate of 95.1% and specificity of 95.9%. This method involves preprocessing noisy ECG signals, employing wavelet analysis and utilising an ARM processor-based feature extraction technique for the detection of QRS complexes and subsequent support vector machine classification. Raspberry Pi 3-based device introduced by Valliappan et al. (11) serves as a bridge between sensors and a mobile ECG app. The device employs a precise peak detection algorithm for real-time analysis with over 95% accuracy. It effectively handles signal processing intricacies such as baseline wandering and power line noise removal while sampling ECG signals at 100 samples per second and transmitting data via WiFi.

Ramkumar et al. (12) discuss the difficulties in detecting atrial fibrillation and point out that there's a lot of variation in who gets screened, how they're monitored and the types of devices used. This variation happens because different people have different levels of risk for AF. For instance, Holter monitoring is often used for people at higher risk of stroke, while portable ECG studies might include healthier individuals. Even though intermittent monitoring sounds promising, there isn't a standard way to do it and it's hard to measure how much AF someone has. Using a single-lead portable ECG has challenges as the algorithms used to detect AF aren't always reliable. Ahmed et al. (13). developed an affordable and user-friendly heart monitoring system that analyses ECG signal variations, comparing them to normal heart rates and RR intervals for early detection of potential AF. Designed for individuals with cardiovascular disease or high-risk factors, the device employs a straightforward yet effective algorithm and digital filtering methods for improved diagnosis of cardiac pathologies.

Malepati et al. (14) designed a prototype aimed at real-time classification of ECG signals utilising an ECG sensor coupled with Raspberry Pi. The system integrates a trained SVM algorithm to detect various arrhythmias based on extracted features with an accuracy of 72.41% for testing data. Diamantino et al. (15) introduce a handheld dual-electrode stick as a portable atrial fibrillation screening device (AFSD) featuring light indication of irregular rhythm and single-lead ECG recording and achieved 90.2% sensitivity and 84.0% specificity for AF detection. This study evaluates its performance in primary care patients referred for echo-cardiograms. Ahsanuzzaman et al. (16) devised a system architecture tailored specifically for detecting atrial fibrillation, employing Long Short-Term Memory (LSTM) techniques for processing raw ECG signals. AD8232 single lead ECG sensor captures heart voltage, with signals processed by Arduino UNO and Raspberry Pi 3 and transmitted to a mobile application via the HC-05 Bluetooth module. Falaschetti et al. (17) discussed a classification algorithm based on recurrent neural networks directly operating on ECG data.

Sowmya et al. (18) employs a CNN-LSTM deep learning model. With a training and validation accuracy of 97.3% and 97.0%, respectively, the model outperforms a standalone CNN. Liu et al. (19) introduced a novel Aggregation Attention Multilabel Electrocardiogram classification model, designed to identify cardiac abnormalities through the utilisation of raw images. Rawal et al. (20) proposed two different 1D CNN Architectures for classification of ECG Signal one with very high accuracy Supreme CNN Architecture (SCA) and another one with low computational power accuracy Software-Selected CNN Architecture (SSCA). Hao et al. (21) presented a filter design methodology aimed at enhancing the quality of ECG signal, specifically targeting R-peak detection, while accommodating constraints imposed by limited hardware resources. The approach strives to yield filters characterised by both low computational complexity and heightened prominence in R-peak detection. Utsha et al. (22) designed a smartphone application with embedded system for continuous ECG monitoring, Heart Rate display and cardiac abnormality detection through edge computing utilising a pre-trained deep-learning classifier.

The evaluation of various approaches to ECG monitoring and early arrhythmia detection has demonstrated the effectiveness of several systems, including Android applications, portable devices, and Raspberry Pi 3 solutions, all of which have different features and degrees of accuracy. Issues like as signal quality during wireless transmission and consistency in monitoring techniques have highlighted the importance of continued advancement in these sectors. Furthermore, the development of advanced classification models, such as CNN and LSTM approaches, suggests a good trend in improving the precision and effectiveness of arrhythmia detection systems. The proposed Integrated Portable ECG Monitoring System with CNN Classification has the potential to be a complete early arrhythmia diagnosis approach that blends cutting-edge technology with practical clinical requirements. This technology provides a cost-effective option for continuous ECG monitoring. It employs CNNs to categorize heartbeats. Its ability to detect arrhythmias early on provides valuable insights into cardiovascular health and encourages collaboration and improvements in the field of portable health monitors.

## 3 Dataset

The MIT-BIH Arrhythmia Database (21), utilised in this proposed study, contains 48 half-hour excerpts of two-channel ambulatory ECG recordings obtained from 47 patients assessed by the BIH Arrhythmia Laboratory. Of these recordings, 23 were randomly selected from a pool of 4,000 24 h ambulatory ECG recordings, reflecting a broad mix of inpatients (approximately 60%) and outpatients (about 40%) at Boston's Beth Israel Hospital. The remaining 25 recordings were specifically chosen to capture less common but clinically significant arrhythmias that would not be well represented in a typical random sample. The dataset was digitised at 360 samples per second per channel with 11-bit precision over a 10 mV range and includes annotations from two or more cardiologists for each record, providing a computer-readable reference for the database's approximately 110,000 beat annotations.

## 4 Proposed methodology

**Figure 2:**
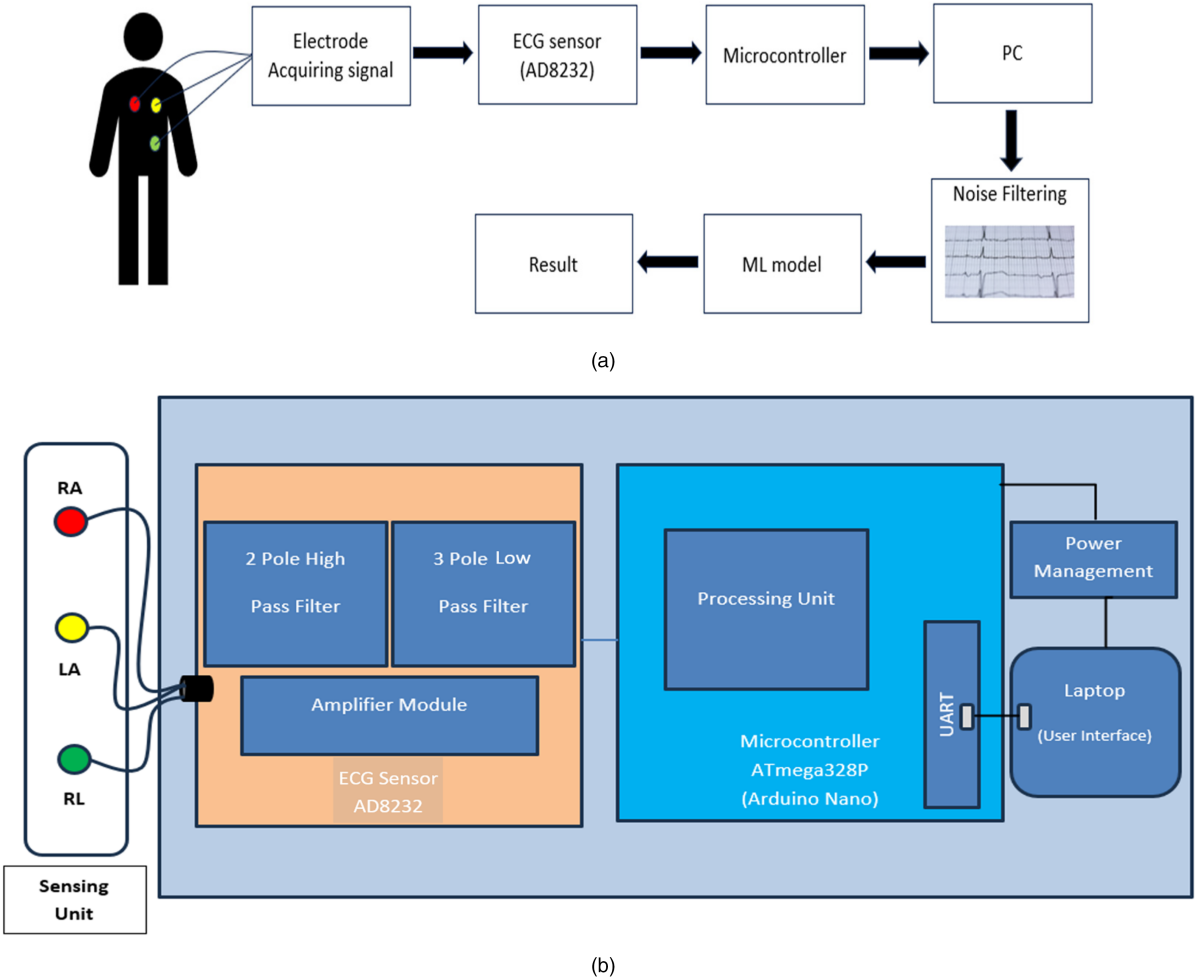
**(a)** Flow diagram of the proposed system: heart signals are captured, processed by the AD8232 sensor, filtered and classified by an ML model. **(b)** Block diagram of the device: heart signals are processed by the AD8232, managed by the ATmega328P and displayed on a laptop interface.

In the proposed work, a portable ECG machine is designed that smoothly integrates standard ECG signal acquisition with cutting-edge machine learning capabilities. The device starts the procedure by detecting electrical signals from the user's body via electrodes put on the chest. The captured signals are then preprocessed, including a filtering step to remove noise and artefacts, resulting in a pure signal ready for further analysis. The sensor converts this electrical activity into voltage variations, which are then sampled by the Arduino Nano at a specified sampling rate. Arduino Nano reads the serial data from the sensor, which contains voltage values corresponding to the ECG signal. These voltage values are then decoded and stored as floating-point numbers in a CSV file for further analysis. The ECG signal data stored in the CSV file is utilised as an input for classification models. Convolution Neural Network models are designed and extensively trained on MIT-BIH datasets containing both normal and pathological heartbeat rhythms. These models function as the core intellect, using its accumulated knowledge to analyse incoming ECG signals in real time. Using complex signal detection, the models accurately distinguish between normal and abnormal heartbeats, providing instant insights into the user's cardiac health. The results of this analytical procedure are immediately transmitted to the user via an interactive interface, delivering real-time data regarding the nature of their heartbeat. This rapid feedback mechanism is extremely useful for monitoring and maintaining heart health. The flow of the proposed work is shown in (Figure 2a).

The block diagram shown in (Figure 2b) illustrates a portable ECG system integrating electrode sensing units [Right Arm (RA), Left Arm (LA), Right Leg (RL)] connected to an ECG sensor with a two-pole high pass filter, three-pole lowpass filter and amplifier module. The sensor data is processed by an Arduino Nano microcontroller equipped with a UART for communication with a laptop, where a machine learning model performs ECG signal analysis for prediction, presenting results through a user interface. The Arduino Nano is chosen due to its small size, low cost and versatility in handling sensor data and running algorithms. AD8232 sensor, designed specifically for ECG signal capture, has high input impedance, low noise and precise signal conditioning capabilities. The built-in instrumentation amplifier guarantees dependable signal acquisition by filtering common-mode interference and amplifying feeble electrical impulses produced by the heart. A buzzer alerts consumers of sudden pulse increases in real time. The user's laptop or mobile device functions as an interface, displaying the classification results. It takes the preprocessed ECG data from the sensor, executes the preset machine learning model for heartbeat categorization, and effectively displays the findings to the user. This configuration allows for compact and portable real-time ECG monitoring and analysis with predictive capabilities.

We provide a portable ECG monitoring system that includes major hardware components optimized for signal capture and user interaction. Our system is built around the AD8232 ECG sensor, which provides low-power, high-precision ECG signal capture that is ideal for wearable health monitoring devices. The specialized integrated circuit has a high input impedance, low noise, and strong signal conditioning capabilities, effectively filtering out common-mode interference while amplifying the faint electrical signals produced by the heart. To further improve signal integrity, the system includes a two-pole high-pass filter and a three-pole low-pass filter, which successfully reduce noise and artifacts in ECG data. The Arduino Nano microcontroller complements the sensor by offering a small and adaptable solution for processing the collected data. The Arduino Nano, which is built around the ATmega328P, is well-known for its low cost and appropriateness for embedded healthcare applications. It oversees data processing and transfer via UART, allowing for real-time delivery of ECG data to connected devices. Furthermore, the device has a buzzer that works as a real-time alert mechanism, notifying users of any detected irregularities in heartbeat patterns. This component improves user involvement by delivering immediate feedback, allowing for timely answers to potential health issues and, eventually, improving the entire user experience with cardiac health monitoring.

Table 1 summarizes the components and specifications of the designed system. It describes the models, operating characteristics, size, and cost of the buzzer, Arduino Nano microcontroller, and ECG sensor. The proposed ECG classification model costs $10.5, which is much less than the $58 reported by S. M. Ahsanuzzaman et al. (16). This demonstrates the cost-effectiveness of our technique while still maintaining great classification performance.

**Table 1:** Hardware specifications.

Component	Model Name	Parameters	Size	Cost
ECG Sensor	AD8232	Operating Voltage (VDC) 3.3 Operating Temperature: −40 to 90°C 3 × Electrode Pads Gain 100 & CMRR 80 dB	4 mm × 4 mm	$6
Arduino Nano Atmega328P-AU MCU	R3 CH340	Operating Voltage (VDC) 5 Power Consumption (Watt) 1 Clock Speed 16 MHz Flash Memory 32 KB	18 mm × 45 mm	$4
Buzzer	–	Operating Voltage (VDC) 3.3∼5.5 Frequency (2,500 Hz)	18.5 mm × 15 mm × 5 mm	$0.5
Total			48 × 35 × 10 mm	$10.5

In our analysis, we concentrate on the specific ECG characteristics that are necessary for recognizing supraventricular ectopic beats, ventricular beats, and fusion beats, all of which are represented in the MIT-BIH dataset. While the QT interval is a significant aspect of ECG analysis, it was excluded from our study. This decision is based on the understanding that the target beats can be accurately detected without an in-depth examination of the QT interval, allowing for a more streamlined approach to arrhythmia classification.

A CNN is designed for heartbeat classification in this ECG machine to extract relevant features from the ECG signals without manual feature engineering. CNN's convolutional layers learn to discover relevant patterns, edges, and feature combinations within ECG signal data, which is critical for correct classification. Conv-1D layers are specifically designed to handle one-dimensional sequential data, making them optimal for ECG signal analysis. These layers conduct convolutions along the temporal axis of the ECG data, collecting patterns and fluctuations over time that are critical for comprehending the intricacies of various heartbeat types. Adam optimizer uses two distinct CNN model architectures for categorization. One approach classifies heartbeats as normal or abnormal, whereas the other divides them into five categories: normal, supraventricular ectopic, ventricular ectopic, fusion, and unknown beats.

### 4.1 Binary classification

**Figure 3:**
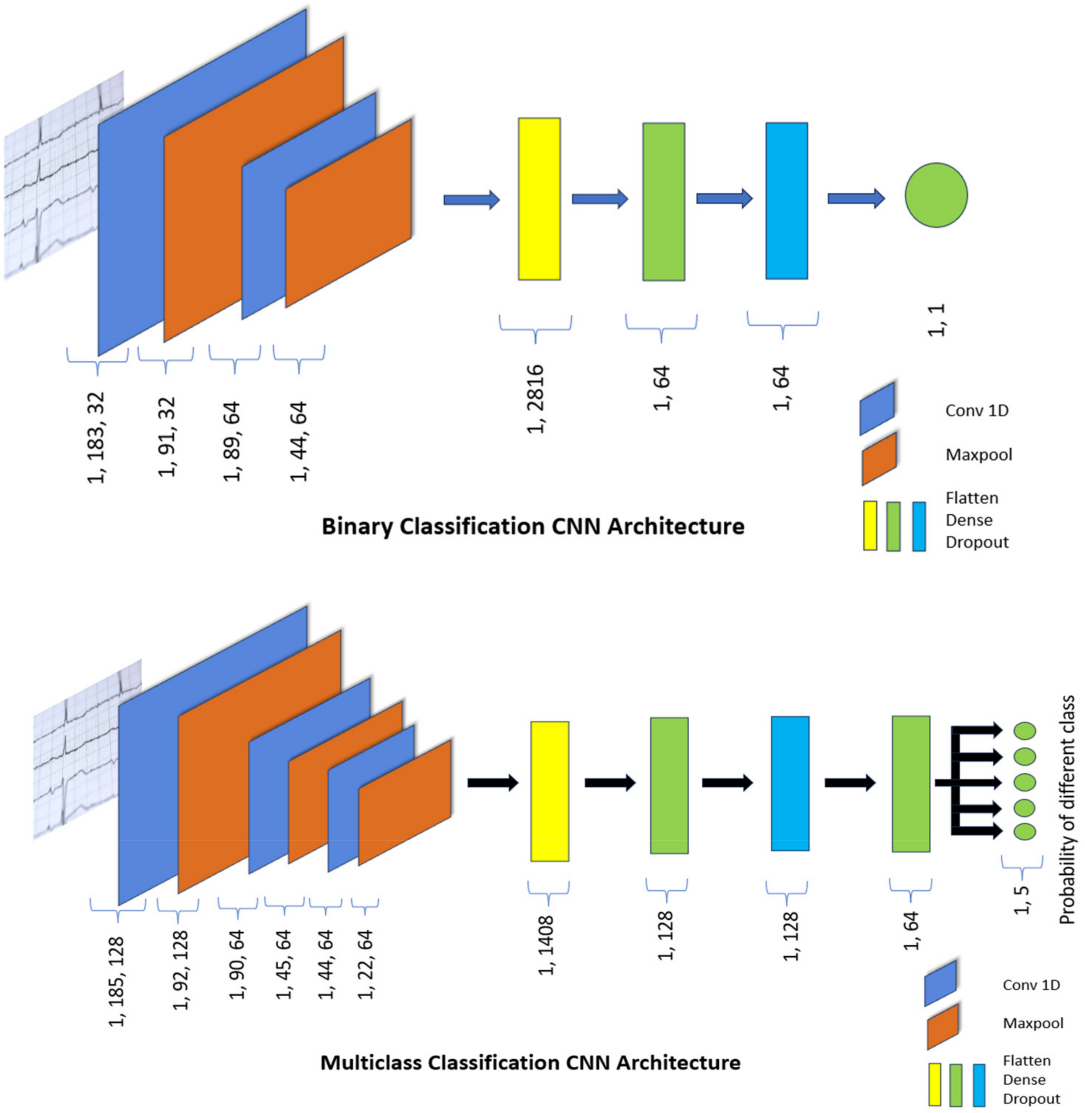
CNN architecture for classification.

Figure 3 illustrates the binary classification neural network architecture, which is composed of up of sequential layers meant to analyze data. It starts with a Conv1D layer with 32 filters and a kernel size of 5, then uses a rectified linear unit (ReLU) activation function to extract various features from the input sequences, which are 187 data points long and have a single feature dimension. This is followed by a MaxPooling1D layer with a pool size of two, which performs downsampling to reduce the dimensionality of the output. Another set of Conv1D and MaxPooling1D layers are provided to capture more detailed patterns and lower the spatial dimensions of the output via the corresponding layers. The Flatten layer subsequently reduces the multidimensional data to a one-dimensional array, which is passed into a Dense layer with ReLU activation. This dense layer enables more advanced feature extraction and representation learning. To avoid overfitting, a Dropout layer is used, which randomly eliminates connections during training to achieve better generalization. Finally, the architecture concludes with a Dense layer containing a single unit and a sigmoid activation function, producing a binary classification output, indicating either normal or abnormal heartbeats based on the sigmoid threshold output. The models' architecture for the binary classification with Layers, Activations, Parameters is give in give in Table 2.

**Table 2:** Architecture details of the proposed binary model for ECG classification.

Layer (type)	Output shape	Param#
conv1d (Conv1D)	(None, 183, 32)	192
max_pooling1d (MaxPooling1D)	(None, 91, 32)	0
conv1d_1 (Conv1D)	(None, 89, 64)	6,208
max_pooling1d_1(MaxPooling1D)	(None, 44, 64)	0
flatten (Flatten)	(None, 2,816)	0
dense (Dense)	(None, 64)	1,80,288
dropout (Dropout)	(None, 64)	0
dense_1 (Dense)	(None, 1)	65
		Total params: 1,86,753 (729.50 KB) Trainable params: 1,86,753 (729.50 KB) Non-trainable params: 0 (0.00 Byte)

Several hyperparameters are selected to optimise performance for the binary classification. The model is trained for 40 epochs with a batch size of 32, allowing it to effectively learn from the data while updating weights in manageable increments. To avoid overfitting, a dropout rate of 0.5 is employed, randomly deactivating half of the neurons during training, which helps the model generalise better to unseen data. The model is compiled using the Adam optimiser, known for its adaptive learning rate capabilities and binary crossentropy as the loss function, which is appropriate for binary classification tasks. This ensures efficient training and accurate performance evaluation.

### 4.2 Multiclass classification

For multi-class classification, we designed a sophisticated and computationally efficient neural network, as shown in Figure 3. The neural network architecture is designed to analyze sequential data using Conv1D, pooling, and dense layers. It starts with a Conv1D layer with 128 filters and a kernel size of 3, which is activated by a rectified linear unit (ReLU) to process data sequences of similar lengths. Following the initial convolution, a MaxPooling1D layer with a pool size of 2 decreases dimensionality while emphasizing important characteristics. Subsequent layers include two Conv1D layers with various filter and kernel sizes, as well as two MaxPooling1D levels for feature refinement. The Flatten layer reshapes the output into a one-dimensional array that the dense layers can process. The architecture continues with a Dense layer of 128 units activated by ReLU to capture higher-level characteristics. A Dropout layer reduces overfitting by randomly deactivating connections during training. Another dense layer, with 64 units and ReLU activation, helps to refine the learned representations. Finally, a Dense layer with 5 units and a softmax activation function helps in multi-class classification by predicting probabilities for five separate classes. This design supports extensive sequential feature extraction, hierarchical representation learning, and multi-class classification across a variety of datasets. Table 3 shows the model architecture for multi-class classification, which includes layers, activations, and parameters.

**Table 3:** Architecture details of the proposed multi class classification model for ECG classification.

Layer (type)	Output shape	Param#
conv1d_2 (Conv1D)	(None, 185, 128)	512
max_pooling1d_2 (MaxPooling1D)	(None, 92, 128)	0
conv1d_3 (Conv1D)	(None, 90, 64)	24,640
max_pooling1d_3 (MaxPooling1D)	(None, 45, 64)	0
conv1d_4 (Conv1D)	(None, 44, 64)	8,256
max_pooling1d_4 (MaxPooling1D)	(None, 22, 64)	0
flatten_1 (Flatten)	(None, 1,408)	0
dense_2 (Dense)	(None, 128)	1,80,352
dropout_1 (Dropout)	(None, 128)	0
dense_2 (Dense)	(None, 64)	8,256
dense_3 (Dense)	(None, 5)	325
		Total params: 2,22,341 (868.52 KB) Trainable params: 2,22,341 (868.52 KB) Non-trainable params: 0 (0.00 Byte)

Several hyperparameters have been selected to strengthen the efficiency and ability to classify different types of heartbeats. The training phase lasts 50 epochs with a batch size of 32, giving the model numerous chances to learn while maintaining computational efficiency. To reduce overfitting, a dropout rate of 0.5 is used, ensuring that the model retains its generalization capabilities. The model is built utilizing sparse categorical cross-entropy as the loss function, which is appropriate for multi-class scenarios with integer-encoded labels. Using Adam optimizer, which allows for dynamic modifications to the learning rate, resulting in enhanced stability and convergence throughout training.

## 5 Results and discussion

This section gives a comprehensive evaluation of binary and multi-class classification using a CNN model for arrhythmia detection, with a focus on performance across diverse arrhythmia types. The CNN model has excellent performance measures, including high recall rates and precision scores, demonstrating its ability to accurately recognize arrhythmia. Furthermore, it emphasizes the CNN model's potential impact on clinical practice and patient care by providing a thorough analysis and demonstrating its applicability and dependability for accurate arrhythmia diagnosis.

### 5.1 Hyperparameter optimization and training dynamics

**Figure 4:**
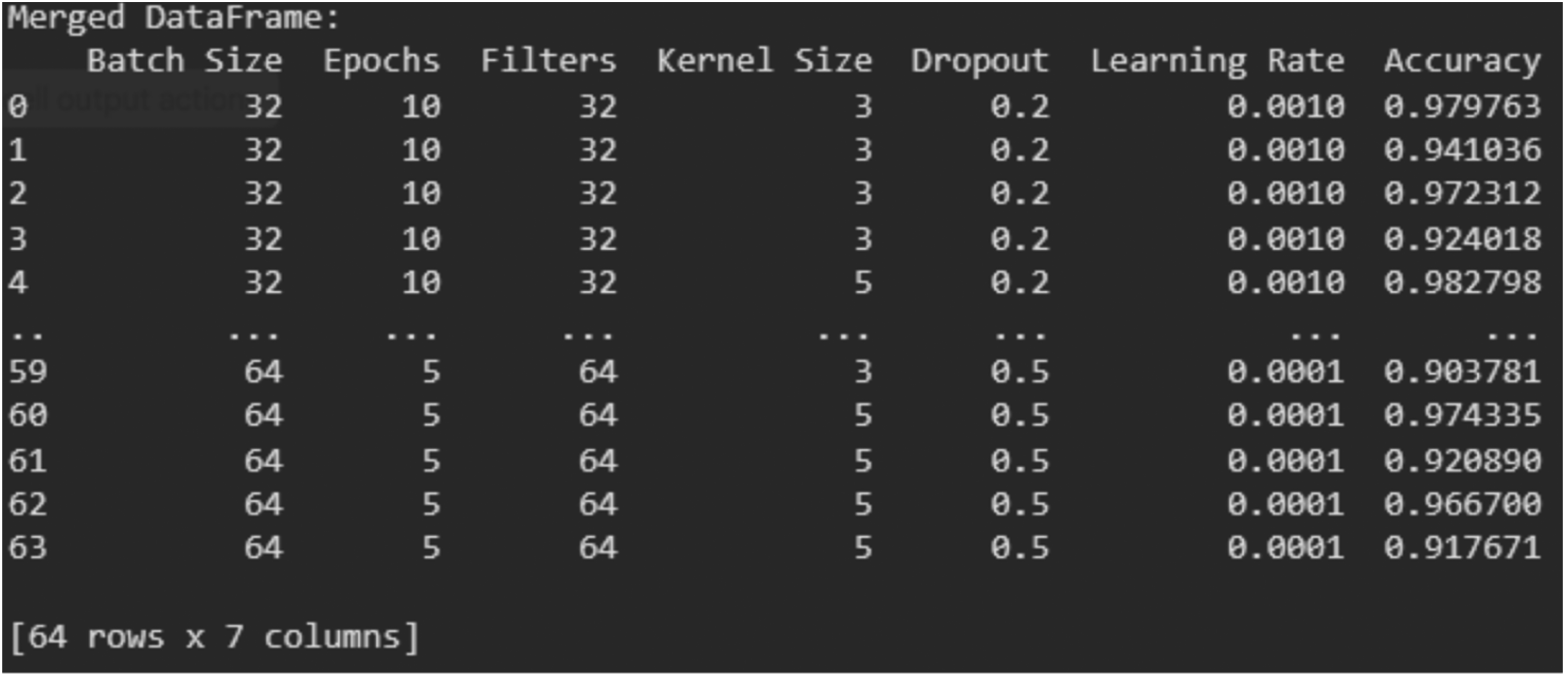
Performance analysis of Various hyperparameter configurations in ECG classification.

An detailed evaluation of different configurations and their impact on model performance was performed to establish the ideal hyperparameters for ECG signal categorization, as illustrated in Figure 4. Based on the results, a two-layer Conv1D architecture was developed, with the first layer using 32 filters with a kernel size of 5 to capture broad temporal patterns in ECG data and the second layer using 64 filters with a kernel size of 3 to extract finer details. This steady increase of filters is consistent with CNN's best practices for feature extraction. A dropout rate of 0.5 was chosen since it gave better generalization than 0.2, hence preventing overfitting. The learning rate of 0.001 consistently beat 0.0001, resulting in stable and efficient convergence.

For training stability and performance, a batch size of 32 was chosen to strike a balance between generalization and computational efficiency. Furthermore, increasing the number of epochs to 20 enabled the model to properly acquire ECG patterns while avoiding the underfitting seen in tests with 5 or fewer epochs. These options were confirmed as the best-performing setup for ECG classification via rigorous hyperparameter tuning and analysis.

Table 4 summarizes the CNN model's performance metrics across several arrhythmia classes in a multi-class classification task. It demonstrates the model's ability to effectively identify cases of each arrhythmia type, as seen by high recall rates ranging from 97.96% to 100%. Furthermore, the model has strong specificity values above 99% in most classes, as well as good precision scores ranging from 98.72% to 99.78%. Low false discovery rates, ranging from 0.15% to 1.99%, demonstrate the model's capacity to reduce misclassifications. With F1 values ranging from 98.42% to 99.84%, the table demonstrates the CNN model's strong performance in reliably diagnosing diverse arrhythmia types, emphasizing its potential therapeutic utility.

**Table 4:** Performance metrics table for multi class classification.

Arrhythmia category	Recall	Specificity	Precision	False discovery rate	F1-score
Normal Beats	97.96	99.96	98.88	0.15	98.42
Supraventricular Ectopic Beats	99.55	99.48	98.72	1.99	99.13
Ventricular Beats	98.45	99.93	99.78	0.28	99.11
Fusion Beats	100	99.85	98.79	0.58	99.39
Unknown Beats	99.93	99.88	99.76	0.44	99.84

**Figure 5:**
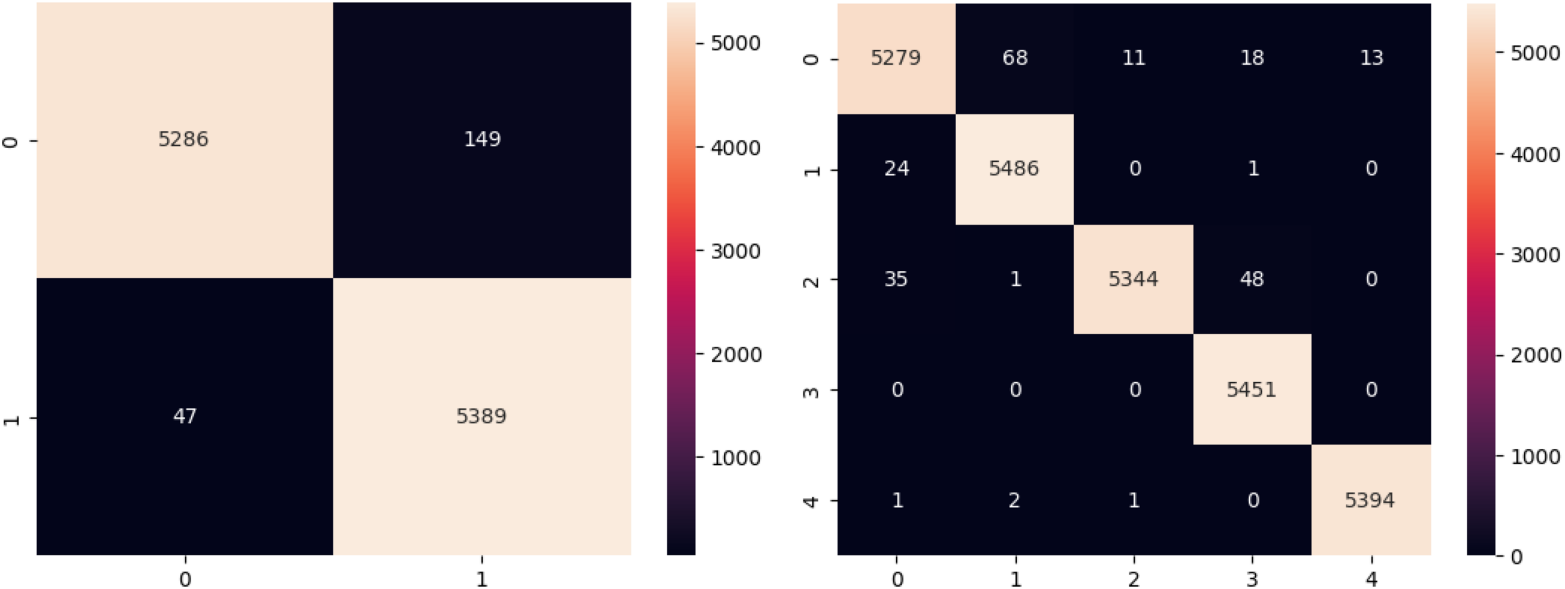
Confusion matrix for binary and multi class classification.

The binary classification confusion matrix given in Figure 5 reveals a strong performance, with 10,675 out of 10,873 instances correctly classified as negative or positive. In the multi-class classification, the model effectively discriminates between five arrhythmia categories, with high values along the diagonal suggesting accurate classification. Despite small misclassifications in some categories, the majority of occurrences are accurately detected, demonstrating the model's accuracy in arrhythmia recognition across various arrhythmia types.

**Figure 6:**
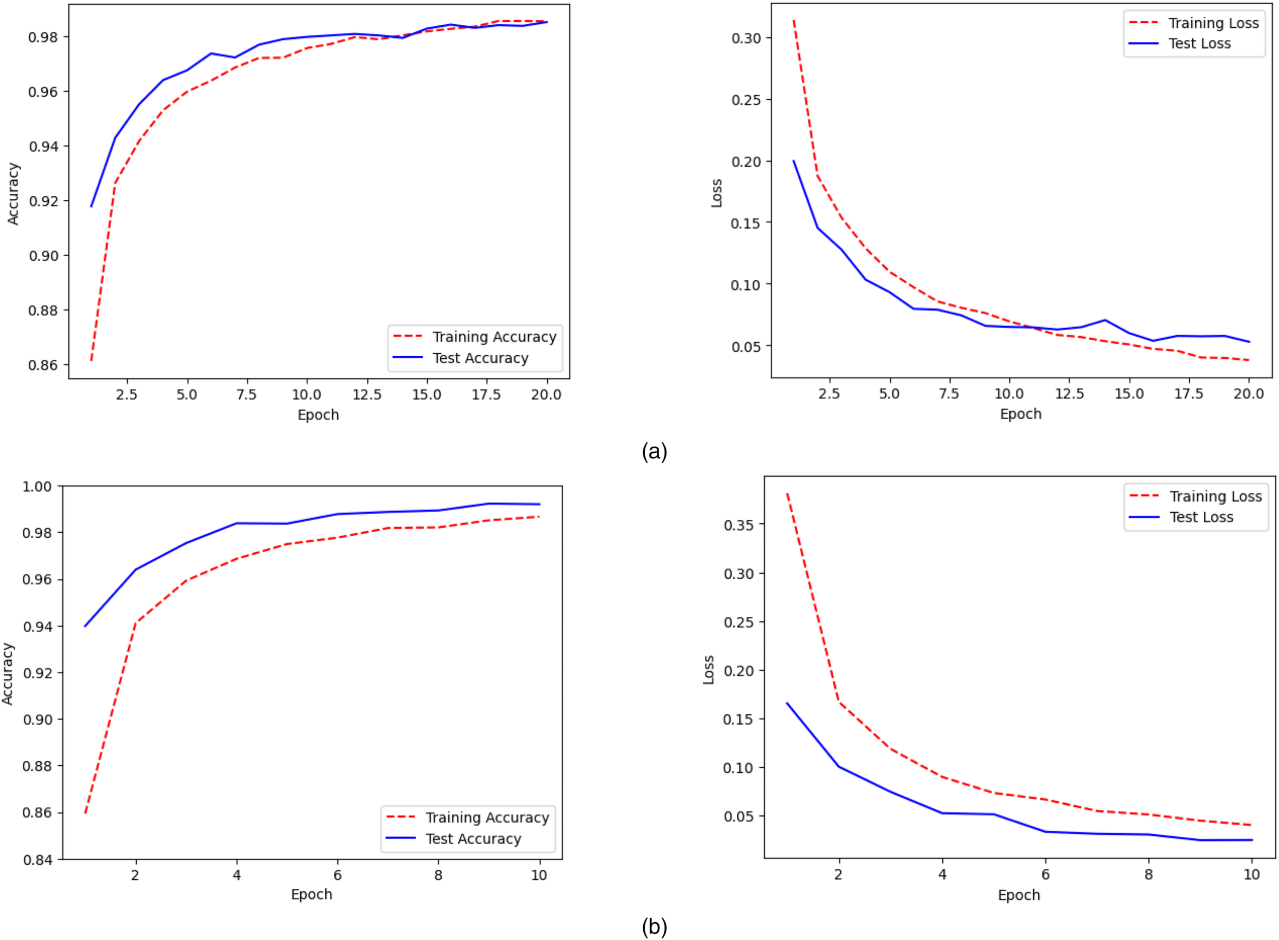
Accuracy and loss plots for **(a)** binary and **(b)** multi class classification.

Figure 6 depicts the Accuracy v/s Epoch plot, which shows how the accuracy of both the binary classification CNN model and the multiclass classification model changes throughout training epochs. It shows the model's learning progress, with higher accuracy signifying better performance over successive epochs. The Loss v/s Epoch in Figure 6 shows how the model's loss function evolves throughout training epochs. Loss values that are decreasing indicate that the model is getting closer to its ideal parameters, which reflects higher performance.

**Figure 7:**
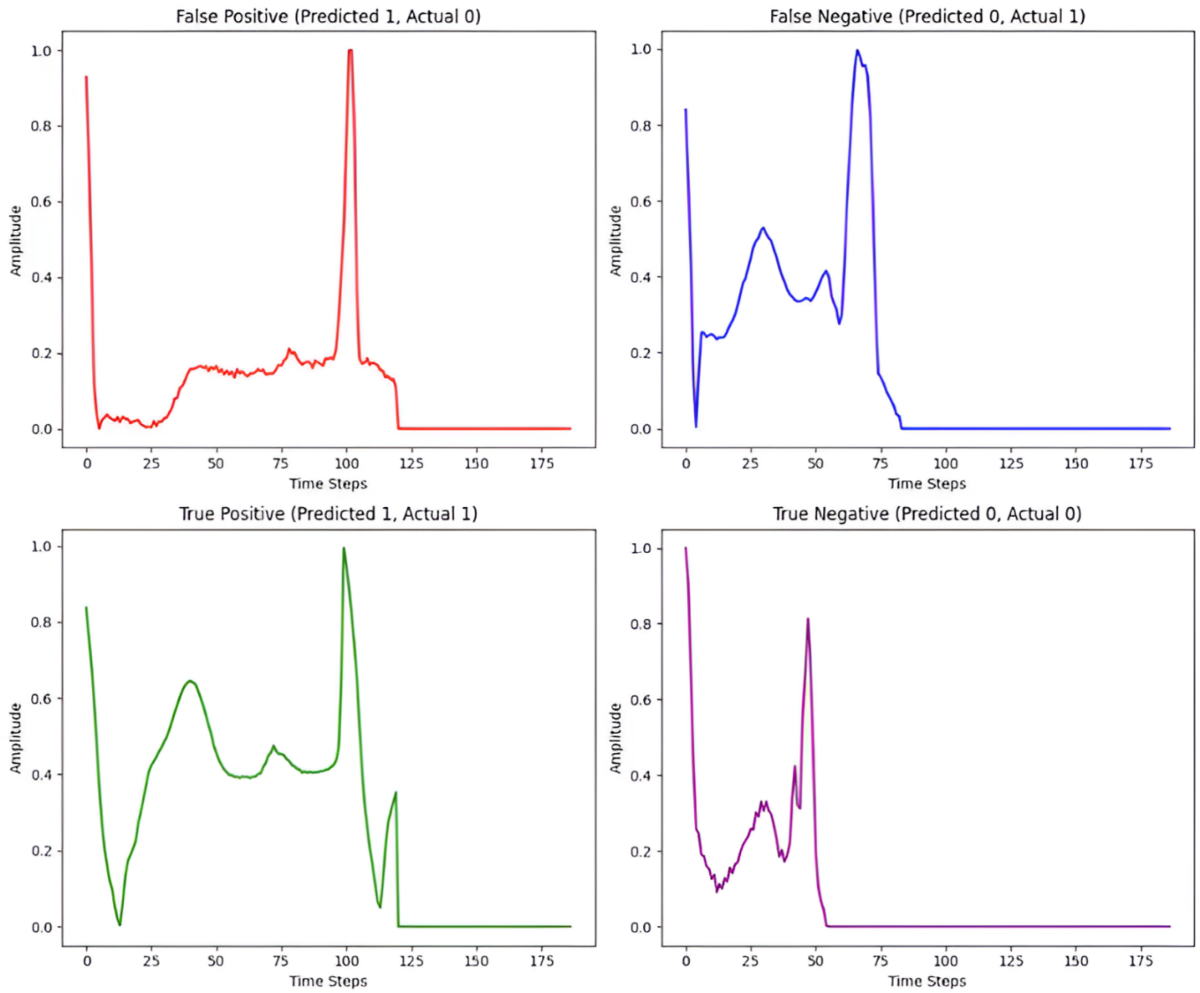
Illustrative ECG signals for different classification outcomes.

Assessing the ECG classification results shown in Figure 7 reveals details about the model's performance and the nature of its faults. For False Positives (FP), where normal beats are misclassified as abnormal, the model is too sensitive to tiny alterations in the ST segment or QRS complex. This sensitivity is represented in the False Discovery Rate (FDR) for normal beats, which, while low at 0.15%, highlights the occurrence of false alarms produced by normal changes perceived as abnormalities. False Negatives (FN) arise when true aberrant beats are classified as normal while being faint, such as mild QRS widening or small alterations in the ST segment. This is evident in the recall scores for several arrhythmia types, with supraventricular ectopic beats and ventricular beats earning 99.55% and 98.45% recall, respectively. While these high values indicate great performance, they also reflect the model's occasional difficulty spotting borderline situations with less obvious problems. On the other hand, the model performs well in recognizing True Positives (TP) and True Negatives (TN). For example, all arrhythmia groups have F1-scores that exceed 98%, with fusion beats at 99.39% and unknown beats at 99.84%. These findings show that the model effectively separates evident examples of abnormality, such as strong QRS widening and distinct ST deviations, while maintaining high specificity (up to 99.96% for regular beats), resulting in consistent rhythm classifications for normal ECG patterns.

### 5.2 Comparative analysis with existing models

The proposed work performs well compared to other models in Table 5 in terms of classification accuracy for both binary and multi-class arrhythmia detection. While the SVM model achieves a binary classification accuracy of 95.9% and the Peak Detection Algorithm reaches 95% for multi-class classification, the proposed 1D CNN models significantly outperform these benchmarks with accuracies of 98.35% and 99.3% respectively. The robustness and high accuracy of the proposed 1D CNN models suggest their efficacy in accurately classifying arrhythmia types, making them valuable contributions to the field of arrhythmia detection and classification.

**Table 5:** Accuracy comparison for different ML models on MIT-BIH dataset.

Ref	Model	Classification	Accuracy
(11)	Peak Detection Algorithm	Multi Class	95.00%
(16)	LSTM	Binary	97.57%
(10)	SVM	Binary	95.9%
(23)	Transfer Learning on pre-trained architecture of DenseNet	Multi Class	98.92%
(24)	2D-CNN	Multi Class	95.20%
(19)	2D-CNN	Multi Class	98.04%
Proposed	1D-CNN	Binary	98.35%
Proposed	1D-CNN	Multi Class	99.3%

### 5.3 Practical implications: efficiency, reliability, and cost-effectiveness

While the proposed system achieves high performance parameters, it also has significant advantages over state-of-the-art systems in terms of computational efficiency, clinical reliability, and cost effectiveness.

• **Computational Efficiency:** The Arduino Nano, powered by the ATmega328P microcontroller, processes ECG data with low computing cost, resulting in real-time performance. Unlike systems based on more resource-intensive platforms, such as the Raspberry Pi 3 (16), our architecture retains efficient processing with much lower power consumption (1 W), allowing for longer operational periods without sacrificing performance.
• **Clinical Reliability:** The AD8232 sensor, with its high input impedance, low noise, and robust signal conditioning, provides precise ECG signal capture. This clinical dependability is equivalent to conventional systems, but it has improved noise filtering *via* integrated two-pole high-pass and three-pole low-pass filters. The real-time feedback mechanism, which includes a buzzer for quick irregular heartbeat notifications, improves user safety and prompt response.
• **Cost-Effectiveness:** The proposed ECG classification model costs about $10.5, which is significantly lower than the 58 USD reported by S. M. Ahsanuzzaman et al. (16). This large cost decrease is accomplished without sacrificing performance, making the system extremely accessible for resource-constrained environments while maintaining excellent classification accuracy.

Overall, the proposed Methodology stands out for its new methodologies and ability to handle variable-length ECG signals while keeping their temporal integrity, resulting in increased feature extraction and accuracy. This approach effectively analyzes sequential ECG data, retaining temporal features through convolutional layers while allowing for faster feature extraction and classification, resulting in improved accuracy. The solution functions in real time, acquiring signals from the ECG sensor at a baud rate of 9,600. The total time required to categorize the ECG signal is roughly 173 ms for binary classification and 218 ms for multi-class classification, indicating that they are appropriate for portable health monitoring systems where prompt alarms are critical for patient outcomes. High accuracy rates reflect the models' ability to effectively categorize ECG signals. Furthermore, it has a real-time monitoring interface that improves user involvement. Comparative assessments of existing solutions show that our technique not only increases classification performance.

## 6 Conclusion

Our proposed portable ECG equipment, which has machine learning (ML) capabilities, stands out for its low cost, ease of use, and high accuracy. Our device's size and cost are rigorously optimized to enable portability while remaining affordable. Its plug-and-play functionality makes it simple to use even for people with little technological understanding. In terms of accuracy, our ML models for ECG signal classification outperform the competition, with 98.35% accuracy for binary classification and an amazing 99.3% for multiclass classification. Our classification models' architecture is highly adjusted to extract key elements from ECG data, resulting in excellent accuracy. Convolutional layers in the binary model identify essential patterns such as QRS complexes and T waves, whereas max-pooling levels lower spatial resolution and noise. The flattened layer condenses these multidimensional features into a succinct format that allows the dense layer to find essential patterns in the flattened feature vector, providing flexibility for capturing intricate relationships within the data. Dropout layers reduce overfitting while ensuring robustness. Similarly, the multiclass model uses a similar architecture but adds additional layers to allow it to distinguish between different sorts of classes. These architectural decisions enable our models to comprehend complicated correlations within ECG data, yielding precise and consistent classification results.

ECG signals, while useful for detecting arrhythmias, are prone to noise and interference from muscle movements and other electrical sources. While filtering techniques help to address these difficulties, attaining absolutely clean signals remains a challenge, potentially affecting diagnostic accuracy. This constraint, however, creates opportunity for future system expansion. Advanced noise reduction algorithms, such as adaptive filtering or deep learning-based systems, may dramatically increase signal clarity. Furthermore, adopting cloud connectivity has numerous benefits. Cloud-based data storage allows for long-term ECG surveillance, while remote monitoring features allow healthcare providers to successfully treat patients with chronic illnesses regardless of location. This continuous data stream delivers real-time information, enabling preemptive intervention and individualized care. To improve therapeutic trust, enhanced interpretability techniques such as SHAP or Grad-CAM could give clinicians with clear explanations of the model's decision-making process. Finally, optimizing the system for real-time performance, which includes lowering inference latency and increasing energy efficiency, is critical for continuous monitoring in resource-constrained, portable contexts.

To enable replication and additional research, we have made the source code public at: https://github.com/Arnavarya35/ECG.

## Data Availability

The original contributions presented in the study are included in the article/Supplementary Material, further inquiries can be directed to the corresponding author.
